# Grounding psychosis research: why observable signs should anchor biological investigations

**DOI:** 10.3389/fpsyt.2026.1777099

**Published:** 2026-02-27

**Authors:** Lena Palaniyappan

**Affiliations:** 1 Douglas Mental Health University Institute, Department of Psychiatry, McGill University, Montreal, QC, Canada; 2 Department of Psychology, McGill University, Montreal, QC, Canada; 3 Department of Psychiatry, Western University, London, ON, Canada

**Keywords:** epistemology, network psychopathology, nosology, phenomenology, psychomotor signs, RDoC, schizophrenia, thought disorder

## Abstract

Biological psychiatry faces a significant epistemic challenge in identifying valid objects for mechanistic research. Both diagnostic constructs and individual symptoms are abstract symbols defined circularly within a closed hermeneutic system, creating a symbol grounding problem that hinders the discovery of biophysical substrates (biomarkers). I argue that progress requires an epistemological separation between the ungrounded symptoms such as delusions and hallucinations, which are co-constructed through personal and clinical interpretation from *grounded signs* that are directly observable features anchored in shared sensorimotor reality. I propose that a Minimal Grounding Set (MGS) can be recovered from the commonly used criteria for psychosis. This MGS, exemplified by disorganization and impoverishment, offers a privileged pathway for the neuroscientific inquiry of psychosis. In this case, (1) biological correlates will be most replicable for MGS than other symptoms; (2) MGS will serve as modular anchors in symptom networks; and (3) progress in precision medicine programs like Computational Psychiatry and quantitative psychopathology frameworks such as hierarchical taxonomy (HiTOP) will depend on explicitly separating MGS from ungrounded symptoms. This approach of *sign-first psychiatry* will provide a non-circular foundation to tether abstract constructs affiliated with psychosis to biological realities that have eluded us for long.

## Introduction: psychosis and the problem of psychiatric objects

What are the most appropriate psychiatric objects to investigate the mechanistic and causal processes underlying psychosis? Diagnostic constructs such as schizophrenia as the objects of study have fallen out of favor due to concerns about validity. Emerging alternatives focus on individual symptoms, investigating them as latent clusters [e.g., HiTOP (1)], dynamic network systems (2), or computationally modeled phenomena (3). This resurgence of symptoms as objects of causal inquiry demands we ask: Are all symptoms of psychosis created equal? The answer is a resounding ‘No’.

Common to all unsuccessful efforts to identify privileged symptoms of psychosis—from Bleuler and Schneider’s ‘first-rank’ systems to the DSM’s fluctuating assignments of importance to various features—is the quest for diagnostic specificity (4, 5). These efforts set out to grant privilege to one level of psychiatric objects (symptoms) based on their purported association with another (diagnosis), with neither grounded on concrete external reality. Like every other complex construct, psychosis faces what Hanard called a *symbol grounding problem (*6). Psychiatric objects such as diagnoses operate as abstract symbols whose meaning is circularly defined by other symbols (e.g., symptoms). This is much like a dictionary that uses Chinese words to define other Chinese words^1^. An outsider with no knowledge of Chinese needs some starting points grounded in sensorimotor reality (e.g., pictures or objects) shared with Chinese speakers, to make a meaningful entry into this symbol system. We face an infinite regress if we attempt to explain a symbol with another. To escape this circularity, at least a small subset of symbols need to be grounded in concrete ‘external’ reality that is accessible to an onlooker’s sensorimotor processes. Such a minimal grounding set (MGS), defined by its bypassing of the hermeneutic process, can then help us to ascertain the biophysical substrates of psychosis (see **Box 1**: Key Terminology).

Box 1 **Key terminology.**

**Minimal Grounding Set (MGS):** The smallest set of clinical features that (1) bypass hermeneutic interpretation, (2) are directly observable, and (3) anchor abstract psychiatric constructs in sensorimotor reality.

**Grounded Signs:** Observable features that do not require patient awareness or self-interpretation and can be quantified in behaviorally-anchored terms.

**Ungrounded Symptoms:** Clinical features co-constructed through patient interpretation and clinical sense-making that lack direct anchoring in shared sensorimotor reality.

**Hermeneutic Opacity:** The obscuring of putative biological signals of mental phenomenon through layers of subjective interpretation and semantic construction.

**Semantic Envelope:** The complex amalgamation of cultural idioms, personal narratives, and clinical interpretations that surround inchoate experiences.

**Sign-first Psychiatry**: An epistemological approach to psychiatric research that prioritizes directly observable, grounded signs over self-reported symptoms when investigating biological mechanisms and causal processes.

## Signs, symptoms, and hermeneutic construction

**Figure 1:**
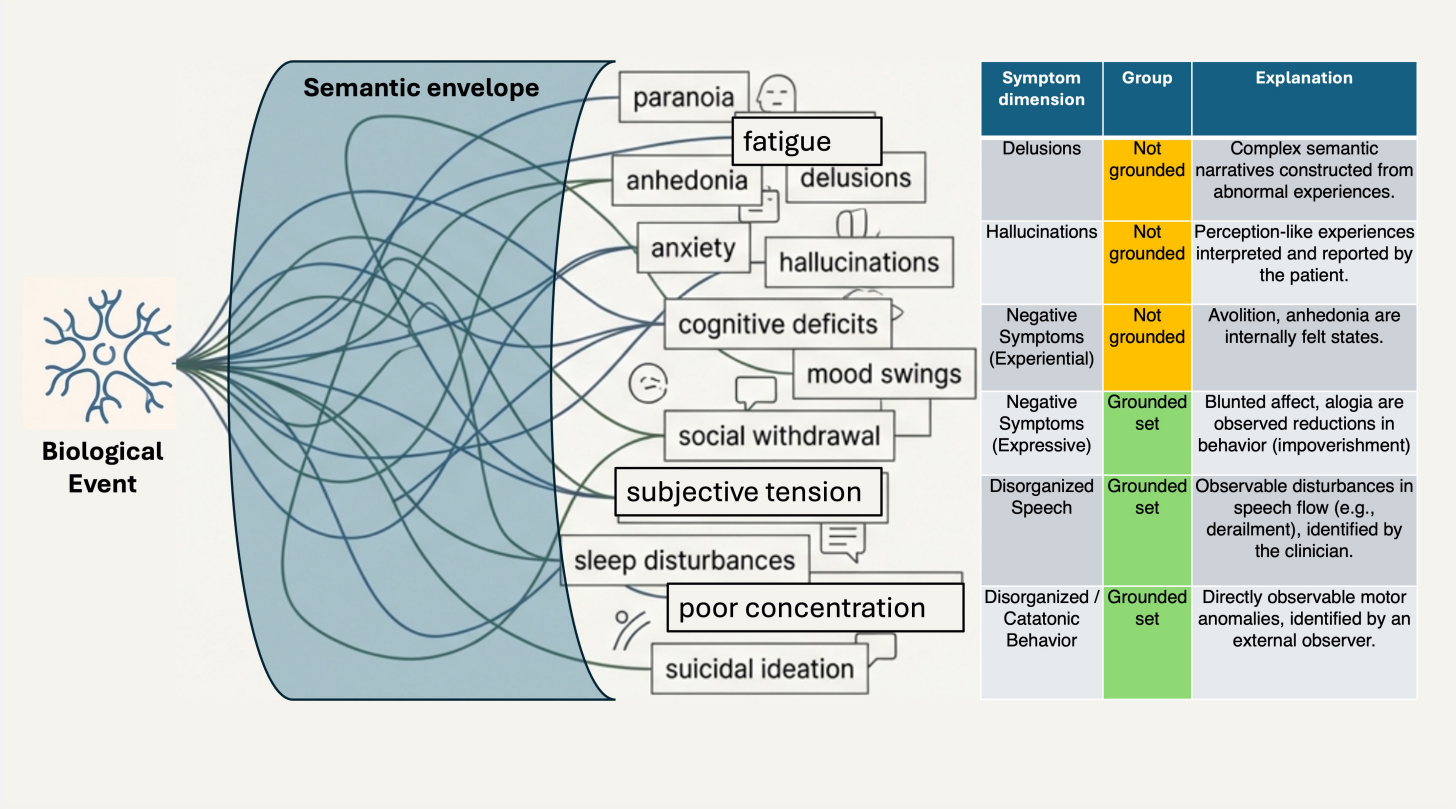
*Left*: Semantic envelope in a patient’s narrative is a co-constructed black-box amalgamating their cultural idioms, background experience, identity as well as clinician’s attempts at sense-making. The resulting labels cannot be mapped clearly to the suspected neural signals. *Right*: DSM-5 schizophrenia criterion A comprises (1) symptoms that are products of complex semantic narratives constructed from abnormal experiences and shaped by personal history and cultural context (e.g., delusions and hallucinations: Not Grounded) and (2) disorganized speech (e.g., derailment, incoherence) and disorganized/catatonic behavior (e.g., posturing)—features where the patient is often unaware of the abnormality but identified by the clinician via direct observation of motor and behavioral anomalies (Grounded set). Negative symptoms show a mixture of both dialogically constructed experiences (e.g., motivational deficit, anhedonia: experiential) and expressed signs (e.g., blunted affect, alogia). Considering the nature of this distinction, we use the term disorganization/impoverishment to describe the observable features. We consider them as the MGS of psychosis, given their grounding in sensorimotor reality of the clinical construct. Parts of this image were created using Google Notebook LM.

In this review, we turn to a model of symptom formation (8) to describe a putative MGS that is most likely tethered to discrete mechanistic processes. Berrios and Markova introduced substantial uncertainty in treating symptoms as straightforward objects of psychiatric inquiry (8). They challenged the inherent assumption that mental symptoms are stable ‘natural kinds’. Instead, symptoms are dialogical entities that exist through an interpretive, co-constructive process between the patient and the clinician. Symptoms generally begin with a neurobiological signal. If its effect penetrates consciousness (the filter of awareness), this creates a raw, inchoate, pre-linguistic experience for the individual who then makes sense of this unfamiliar experience. Individuals configure their inchoate experience using personal narratives, familial idioms, and cultural templates available to them. This is a hermeneutic act of self-interpretation. This interpretation is further shaped via interactions with a clinician, who may help disambiguate or label it, the professional act of clinical sense-making (9). This process of co-construction adds several layers of abstraction, obscuring the link between the original biological signal and the final reported symptom (**Figure 1**).

A person experiencing an ineffable subjective phenomena of disembodied thoughts may report this as ‘inner voices’ or ‘intrusive thoughts’ based on their familiarity with these concepts. We often see the same uncomfortable feeling of being socially judged described as social anxiety by some and paranoia by others. Based on the semantic construction that one chooses to follow, other symptom experiences may also ensue. For example, experiencing disembodied thoughts as ‘voices’ may lead to the experience of anxiety, and building explanations that are labeled delusions. Thus, symptoms that are formed via awareness and constructive interpretation of inchoate signals may further fork up and produce other symptoms, resulting in a network of psychopathology (10).

This hermeneutic process is further complicated in clinical practice, where tools like symptom checklists (heavily used by HiTOP) force further interpretation while clinicians add another layer of complexity via their nosological and probabilistic intuitions. Consider a symptom checklist item such as “my concentration is poor”. This can be endorsed by people with 3 radically different experiences: one person with an inability to generate a continuous stream of thoughts on a specific topic, another who finds initiating thoughts as effortful and thus not sustainable for long, while a third person who is getting easily distracted (11). These represent distinct neurocognitive events with likely different neural bases, yet when introduced to “my concentration is poor”, all three will endorse this item. Clinicians do not label symptoms in a theoretical vacuum; their identification of symptoms is invariably shaped by the very diagnostic categories (or symptom clusters) they seek to validate [e.g., inner voices may be labeled as hallucinations in a clinic for schizophrenia, but as pseudo hallucinations when searching for clues of emotionally unstable personality; distorted body image perception may not be labelled a delusion if it occurs in the context of eating disorders; also see (12)]. Thus symptoms are always symptoms of an illness (13); More crucially, symptom assessment is never atheoretical, leaving the dialogical construction process a hermeneutic black-box; relating its products to biophysical substrates (grounding) is a ‘hit-or-miss’ endeavor.

## Toward a minimal grounding set of psychosis

When we consider this hermeneutic process, we may conclude that many symptoms are irrevocably opaque, shadowing the underlying diagnostic constructs, and cannot serve as reliable anchors for neuroscientific investigation (11). This conclusion, however, overlooks a crucial distinction made by Berrios and Markova who identified another pathway of symptom formation with profound implications for identifying valid objects of neuroscientific inquiry (8). Certain psychiatric objects are observable signs; they also originate from a neurobiological signal but do not pass through the filters of consciousness (i.e., patients are often unaware of exhibiting them), thus bypassing the dialogical interpretations that characterize other symptoms. Given their availability to natural senses i.e., our ability to see, touch and hear them, the role of semantic construction is considerably thinner. Consequently, these features are grounded on sensorimotor reality of the shared world.

Based on these hints, we can define a clinical feature as a constituent of the MGS if (a) it is detectable by an observer’s senses without relying on the patient’s self-interpretation; (b) the patient is not required to hold any meta-representational stance on the experience; (c) its grading can, in principle, be specified in a behaviorally anchored, context-minimal manner (see **Supplementary Figure 1**: Decision Algorithm).

Notably, not all signs can be included in MGS. For example, Russell’s sign of bulimic knuckles (calluses on knuckles due to repetitive induction of vomiting), is likely to be a distal feature of a biological signal that has gone through the hermeneutic construction (body image distortion). A sign, if it represents a secondary consequence of a symptom, cannot be in the MGS.

On the other hand, there is also no requirement that a MGS feature must always be a sign elicited by a clinician. For example, motivational deficit (avolition) is categorized as an experiential negative symptom when it is reported as an internally felt, subjective state. However, if lack of motivation becomes quantifiable using an actigraphy measure that tracks measurable reduction in psychomotor activity, avolition transforms from an ungrounded symptom to a feature of MGS (part of impoverishment). Of note, avolition loads on both experiential and expressive negative symptoms in factor analysis (14).

Applying these principles to DSM-5 ‘criterion A’ symptoms of schizophrenia (**Figure 1**), we recover a set of symptoms—disorganization/impoverishment—as likely candidates for MGS in psychosis.

## Disorganization and impoverishment as grounded signs

Unlike the tangled web of mutually reinforcing delusions and hallucinations, MGS features of disorganization/impoverishment offer discrete, directly observable entry points into the neurocognitive machinery of psychosis. They are unlikely to exhibit causally ambiguous co-occurrence patterns that is often seen among the 3 ungrounded features—delusions, hallucinations and experiential negative symptoms. For example, disorganized speech can co-occur with but cannot logically *induce* catatonic posture; but hallucinations can result in paranoid delusions, which in turn can induce lack of enjoyment in social activities. Furthermore, MGS offers a crucial, normative continuum, connecting pathology back to basic, observable human functions (e.g., speech, affective expression). Ungrounded symptoms, by contrast, require an arbitrary, sharp qualitative break to leap from normality to pathology. Any continuum model for ungrounded symptoms requires them to be different from an expected psychological function in the first place, before we examine their continuous distribution in terms of frequency, distress and disability in the population. In other words, ‘excessive beliefs’ or ‘more frequent perceptions’ are not delusions or hallucinations, while ‘excessive talking’ and ‘more frequent blinking’ are pressured speech and motor stereotypy respectively. Thus MGS are likely to have direct ties to specific neurocognitive functions and their disruptions, free of semantic envelopes, but also assume a distinct modular privilege within network models of psychopathology. The proximity of disorganization to polygenic risk of schizophrenia (15, 16) and impoverishment to autoimmune causal mechanisms provide circumstantial support to our case.

Despite their long history of being used to mark the severity of psychiatric conditions (e.g., hebephrenia in schizophrenia, psychomotor retardation in melancholic depression), psychomotor features that are grounded in the sensorimotor reality of an observer have been underemphasized in modern practice (17), including in definitions of ‘high-risk’ states of psychosis (18). In part this may be due to the fact that many MGS are not diagnostically specific [e.g., psychomotor retardation in schizophrenia and depression (19)] while most high-risk states are defined with a diagnostic construct as outcome. Nevertheless, MGS such as motor abnormalities in youth has demonstrated predictive and discriminative value for broad diagnostic outcomes such as psychosis (20) though the field’s predominant focus on self-reported positive symptoms has led to their de-emphasis.

This exemplifies the broader problem the MGS framework addresses: epistemologically privileged signs that offer more reliable pathways to biological mechanisms have been neglected in favor of hermeneutically complex symptoms that may be less stable and harder to ground in neural substrates. In fact, there is a tendency to use signs and symptoms interchangeably in psychiatry (21). Conflating these two to generate latent factors or diagnostic constructs will continue to impede progress towards biophysical grounding of psychiatric constructs via noisy, inconsistent results. For large-scale initiatives like the Accelerating Medicines Partnership (AMP^®^) Schizophrenia (22), the sign-first approach of MGS that prioritizes disorganization and impoverishment may provide more viable and stable solutions to the questions posed.

## Theoretical justification to implementation challenges

It is important to note that MGS are not simple proxies for ungrounded symptoms. They are independently measurable behavioral anchors whose relationships to other clinical phenomena can be one-to-many; such dependencies can only be established via empirical investigations. For example, actigraphic reductions in motor activity correlate with various clinical phenomena, including apathy (23), parkinsonian signs, catatonic features, and negative symptom severity (24). This diversity (or multifinality) supports the claim that the machine-readable reduction in psychomotor output (the grounded phenomenon) is likely more proximal to the biological signal, while its experiential correlates (avolition, anhedonia, fatigue) vary based on context and individual differences. A person with markedly reduced actigraphic activity may be enjoying all of their low-effort activities, showing that the grounded (impoverishment) and the ungrounded (anhedonia) features are distinct phenomena requiring separate measurements.

**Figure 2:**
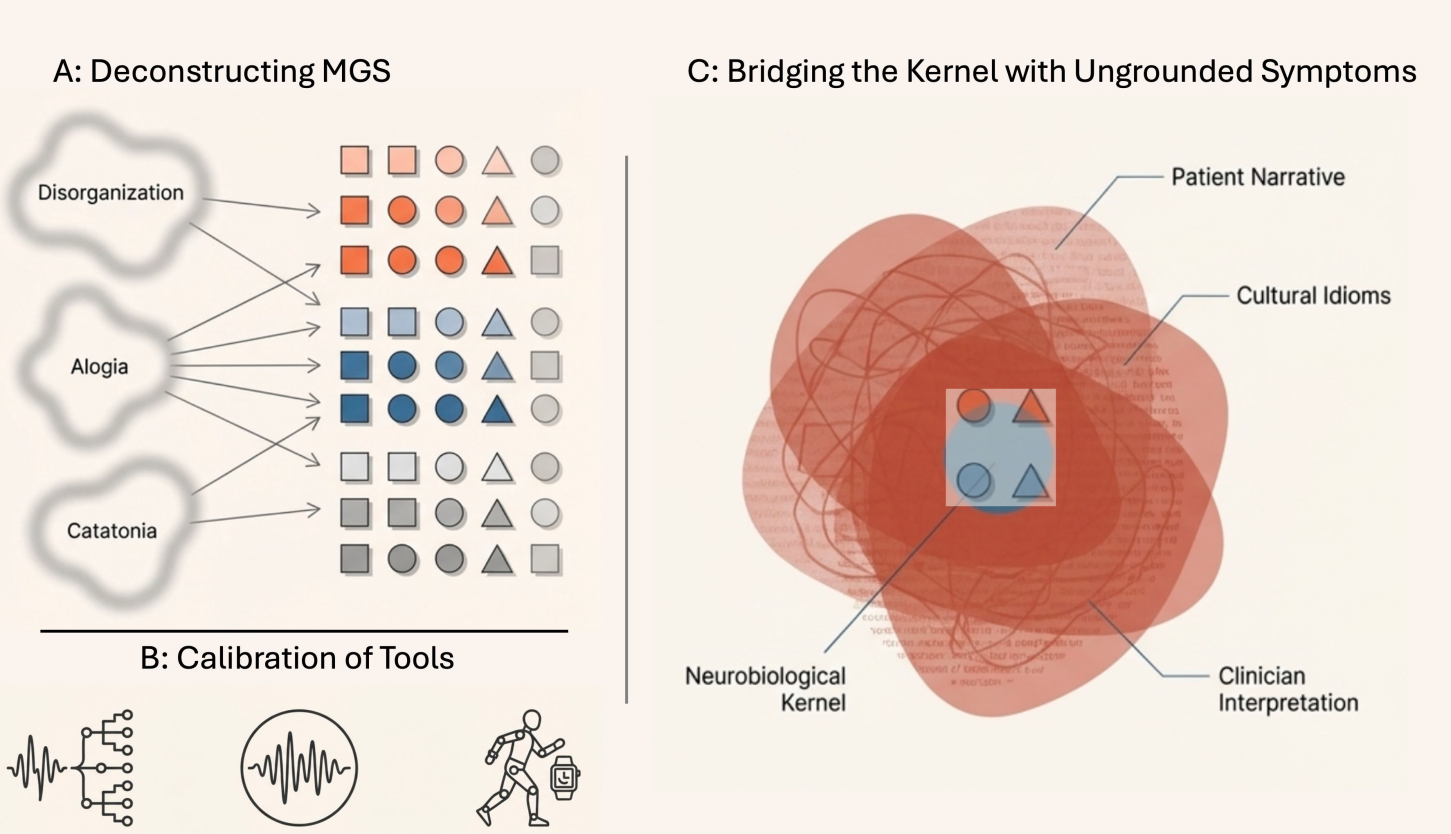
Deconstructing, Measuring, and Integrating the Minimal Grounding Set (MGS) of Psychosis. **(A)** Deconstruction of broad clinical categories (e.g., disorganization, alogia, catatonia) into fundamental, observable signs that constitute the MGS. **(B)** Technology-driven tools for objective quantification of MGS features, such as computational linguistics, acoustic analysis, and wearable sensors, reducing reliance on subjective clinical interpretation. **(C)** Integration of neurobiological substrates mapped to MGS with abstract, ungrounded symptoms (e.g., delusions, hallucinations), constructing a nomological network that bridges sensorimotor signs and hermeneutic experiences in psychosis. Parts of this image were created using Google Notebook LM.

The MGS framework, while offering a promising path to biologically-grounded mechanistic research, faces significant practical barriers that have led to their clubbing with other symptoms and their underutilization in neuroscience. The 3 core problems are:

1. **Symptom heterogeneity**: Broad clinical categories like disorganized speech are not unitary constructs. Specialized rating instruments (formal thought disorder scales) that purport to measure them contain a plethora of items, with minimal overlap among scales. While most of the items in these scales are objective MGS features, many are low-base-rate phenomena: e.g., catatonic posturing or neologisms. They may be highly indicative of specific neurocognitive disruptions, but their infrequent occurrence makes them statistically inefficient targets for large-scale neuroscientific pursuits. An important step in the pursuit of the mechanism of psychosis is deconstructing broad MGS (e.g., disorganization, alogia, catatonia: **Figure 2A**) into their fundamental, constituent features, rigorously identifying the most characteristic objective signs and estimating their prevalence across disorders, stages of illness, and in specific groups. As the features that belong to MGS are not qualitatively differentiated, the issue of rarity is one of sensitivity of our tools in picking the features in varying degrees among the assessed.
2. **Reliability of measurements**: Objectivity of MGS per se does not ensure their reliability in clinical practice. As many behaviors can be subtle and periodic, they may evade clinical detection, unlike the constructed experiences that are recalled at will. Without standardized, behaviorally-anchored definitions and rigorous training, clinicians may inadvertently transform objective data into noisy, subjective ratings. In many cases, psychiatric signs are not passively present (cf., a heart murmur detected with a stethoscope); they must be actively elicited within the context of interviews via appropriate clinical maneuvering. For example, the recommended structure for the evaluation of catatonia exemplifies the best practices needed for other MGS (25): combining systematic observation, strategic interview interactions to actively elicit specific signs, and standardized physical examination to assess muscle tone and other features. Developing, calibrating and applying scalable, technology-driven tools to quantify MGS with minimal context dependence will ensure that their assessment moves to the objective domain (**Figure 2B**). Examples include: Natural Language Processing for quantifying speech coherence, syntactic complexity, lexical diversity, and idea density; Speech Signal Processing for measuring prosody, speech rate, and pause patterns; Wearable Sensor Technologies for quantifying psychomotor activity, gesture, posture, and specific catatonic phenomena. In fact, given their sensorimotor grounding, dense behavioral sampling is much better suited for MGS than for the ungrounded set of symptoms.
3. **The need for thresholds:** While MGS are grounded in continuous behavioral dimensions, clinical practice requires some cut-offs on what constitutes ‘reduced’ expressivity, ‘disorganized’ speech, or ‘abnormal’ motor activity. Such thresholds depend partly on population norms that vary across cultures, age groups, and contexts (e.g., prosodic patterns and gestural expressivity show notable cultural variations). Establishing such thresholds requires collecting normative data across diverse populations to establish appropriate reference ranges and calibrating computational algorithms to account for such variations. Thus technological objectivity does not automatically confer cultural invariance or eliminate the need for normative benchmarking but enables one to capture the full range from subtle alterations in early, prodromal phases to severe manifestations in acute episodes. See **Box 2**: Research Roadmap for the critical steps needed to realize ‘sign-first psychiatry’ via MGS framework.

Box 2 **A research roadmap for MGS framework in ‘sign-first psychiatry’.**

**PHASE 1: Establishing the Foundation**

*• Validate computational tools for MGS quantification*

 – Computational linguistics tools for disorganization (e.g., LLMs to estimate coherence (26))

 – Wearable sensors/kinematics for impoverishment (psychomotor activity, gesture analysis (27))

*• Establish normative datasets across diverse populations*

 – Cross-cultural benchmarks for MGS (accounting for linguistic, and cultural variations (28));

 – Age/sex-stratified norms from adolescence through late adulthood

*• Demonstrate MGS-brain mapping with higher replication than traditional approaches*

 – Multi-site neuroimaging studies testing MGS predictions

 – Compare MGS *vs*. ungrounded symptom associations with neural circuits (29)

 – Examine genetic architecture of MGS features (heritability, polygenic risk scores)

**PHASE 2: Construct Integration**

*• Test MGS as network anchors in models with ungrounded symptoms*

 – Network analysis demonstrating MGS features as modular nodes

 – Test centrality of MGS in symptom networks (see Mülfarth and colleagues (30))

 – Compare MGS *vs*. ungrounded symptom-based network stability across clinical states

*• Examine MGS temporal stability vs. ungrounded symptoms*

 – Stage-specific analysis including prodromal/high-risk states (31)

 – Longitudinal studies of MGS features e.g. ecological momentary assessments

 – Compare state *vs*. trait characteristics of MGS *vs*. ungrounded symptoms

• *Develop MGS-based early detection algorithms*

 – Machine learning models to predict psychosis onset (see Corcoran and colleagues (32))

 – Test efficacy of monitoring systems (smartphone-based speech/activity tracking)

**PHASE 3: Clinical Utility**

*• MGS-guided treatment development*

 – Develop interventions specific for MGS (e.g., behavioral activation for impoverishment)

 – Test whether MGS improvements predict functional recovery

 – Examine MGS as treatment response biomarkers (e.g., see Cohen and colleagues (33, 34))

*• Cost-effectiveness of MGS-based assessment*

 – Compare MGS-guided *vs*. standard clinical assessment on diagnostic accuracy and efficiency

 – Evaluate implementation costs of computational MGS tools in routine clinical settings

## Objections and limitations

The call to focus on MGS does not dismiss the possibility that the experience of some ungrounded symptoms may directly relate to neural substrates circumventing hermeneutic opacity. For example, visual hallucinations occur with occipital stimulation; recurrent obsessions reduce with deep-brain stimulation. In arguing for their importance, I do not claim that MGS is sufficient or necessary for the eventual clinical construct (symbol system) of psychosis; nor do I deny that sensible biological substrates can be uncovered for ungrounded symptoms. I merely position MGS as a privileged starting point—indispensable for bringing non-circular meaning to the construct of psychosis (and by extension, for other diagnostic constructs), as it is amenable for objective quantification while being unhinged from hermeneutic opacity. The neurobiological kernel that is mapped to MGS needs to be subsequently studied in relation to the eventual clinical construct, as well as in relation to the ungrounded abstract symptom set, to build a nomological net around the refined psychosis construct (**Figure 2C**; see **Table 1** for an outline of predictions). The resulting empirical resolution of the psychosis construct may reveal its long-suspected plurality (35); the MGS framework per se is agnostic of the true latent structure.

**Table 1:** Quantifiable predictions of the MGS framework across research domains.

Research domain	Minimal Grounded Set (MGS) features	Ungrounded symptoms
**Biological Psychiatry** *(Neuroimaging, genetics, neurophysiology)*	Stronger, specific, replicable links to neural substrates e.g., Cohen’s d ≥ 0.8 for brain–MGS associations (large effects) with higher replication rates in independent samples with the same methodology.	Weaker, less specific, less replicable links to neural substrates e.g., d = 0.2–0.5 (small to medium effects) for brain–MGS associations with lower replication rates in independent samples with same methodology
**Symptom Networks**	Independent, modular nodes that act as candidate anchors for biophysical parameters with temporal stability, community modularity and cross-sample consistency	Densely interconnected, mutually reinforcing nodes with stronger links to sociocultural context when modeled together
**Computational Psychiatry**	More utility in representing and recovering latent illness states and higher contribution to classification accuracy when including MGS.	Less reliable in the recovery of latent states with classification accuracy when including ungrounded symptoms alone.

Predictions based on MGS for various psychiatric research paradigms. MGS features are expected to show stronger biological correlations, serve as network anchors, and provide more reliable computational targets compared to ungrounded symptoms.

## Conclusion: grounding psychosis in sensorimotor reality

In summary, I argue for an epistemologically informed separation of psychiatric objects of interest. Clinical features rooted in the sensorimotor domain (disorganization and impoverishment) are likely to provide the much needed grounding to identify biophysical substrates of psychosis; once identified, these substrates can strengthen the validity of higher level constructs (e.g., diagnoses, dimensions, stages etc.). Paradoxically, side-stepping from the complexity of dialogical interpretations (i.e., sign-first approach) is the fastest path towards understanding the signals that spark such interpretations in the first place. Separating grounded from the ungrounded feature sets in psychopathology is an epistemological necessity for a meaningful biological psychiatry.

## Data Availability

The original contributions presented in the study are included in the article/**Supplementary Material**. Further inquiries can be directed to the corresponding author.

## Footnotes

1 This analogy was first invoked by the philosopher John Searle (7)

## Supplementary material

Supplementary Figure 1 Decision algorithm for evaluating whether a clinical feature qualifies as part of the Minimal Grounding Set (MGS). Clinical features are evaluated sequentially against five criteria: (1) direct observability by external observers (pressured speech, not anhedonia), (2) independence from patient self-interpretation (alogia, not anxious tension), (3) quantifiability in behaviorally-anchored terms (speech rate, not grandiosity), (4) grounding in sensorimotor reality accessible to others (incoherent speech not referential ideas), and (5) minimal context-dependence in measurement (echolalia not lack of insight). Of note, no measurement is entirely context-free, but we must be able to *assess* MGS features irrespective of cultural background, personal history or social context to identify the phenomenon of interest.
