# Supplementary figures and images for: Grounding psychosis research: why observable signs should anchor biological investigations
Source: Front Psychiatry. 2026 Feb 27;17:1777099. doi: 10.3389/fpsyt.2026.1777099 (PMC12983040; doi:10.3389/fpsyt.2026.1777099)

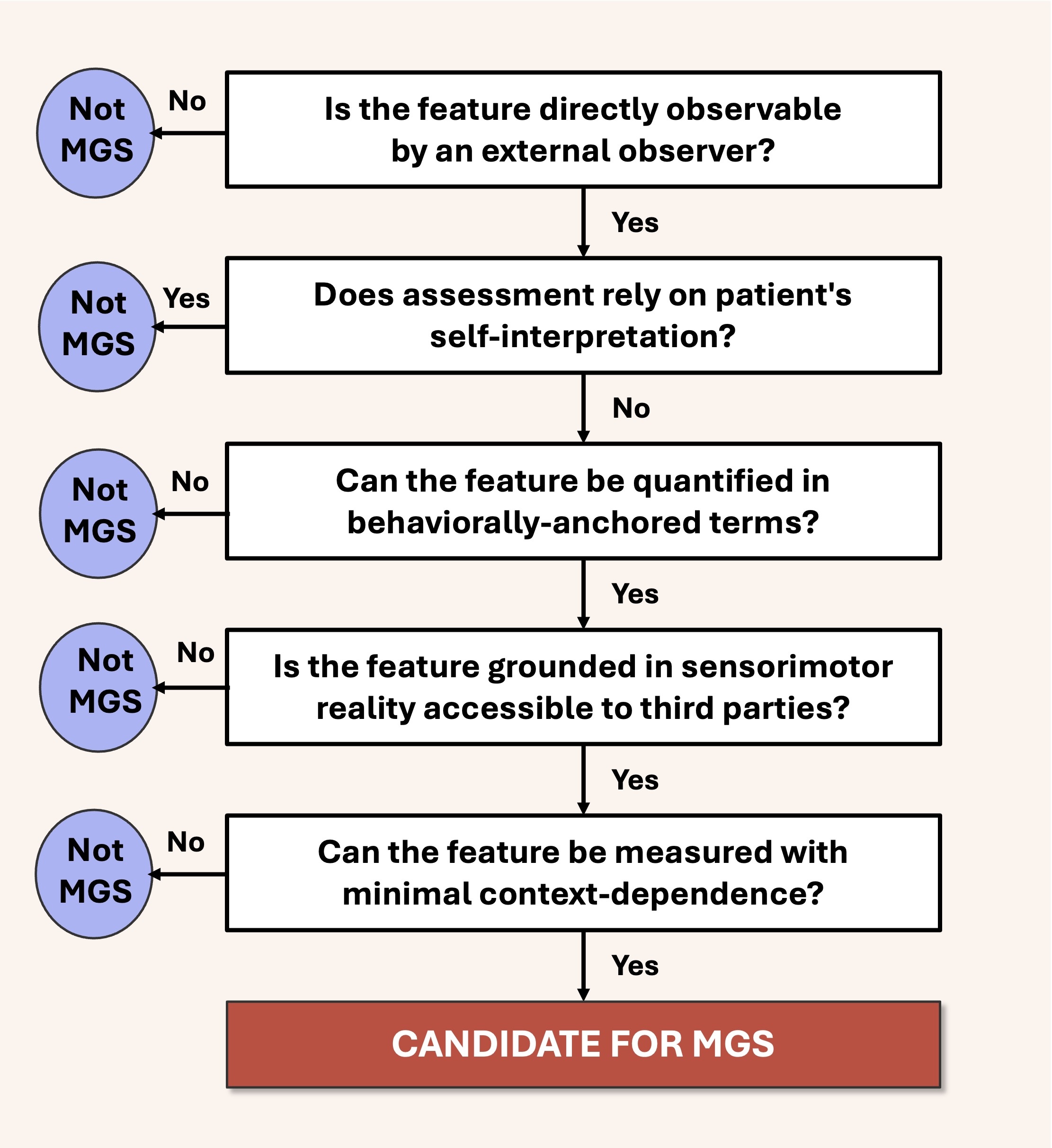

Supplement: Supplementary Figure 1 — Decision algorithm for evaluating whether a clinical feature qualifies as part of the Minimal Grounding Set (MGS). Clinical features are evaluated sequentially against five criteria: (1) direct observability by external observers (pressured speech, not anhedonia), (2) independence from patient self-interpretation (alogia, not anxious tension), (3) quantifiability in behaviorally-anchored terms (speech rate, not grandiosity), (4) grounding in sensorimotor reality accessible to others (incoherent speech not referential ideas), and (5) minimal context-dependence in measurement (echolalia not lack of insight). Of note, no measurement is entirely context-free, but we must be able to assess MGS features irrespective of cultural background, personal history or social context to identify the phenomenon of interest. [file Image1.jpg]
